# Chemical Characterization and Visualization of Progressive Brown Rot Decay of Wood by Near Infrared Imaging and Multivariate Analysis

**DOI:** 10.3389/fpls.2022.940745

**Published:** 2022-07-12

**Authors:** Tiina Belt, Muhammad Awais, Mikko Mäkelä

**Affiliations:** ^1^Production Systems Unit, Biomass Characterization and Properties, Natural Resources Institute Finland, Espoo, Finland; ^2^Department of Bioproducts and Biosystems, Aalto University, Espoo, Finland; ^3^VTT Technical Research Centre of Finland Ltd., Espoo, Finland

**Keywords:** cellulose degradation, *Coniophora puteana*, earlywood, imaging, latewood, lignin modification, near infrared spectroscopy, *Rhodonia placenta*

## Abstract

Brown rot fungi cause a type of wood decay characterized by carbohydrate degradation and lignin modification. The chemical and physical changes caused by brown rot are usually studied using bulk analytical methods, but these methods fail to consider local variations within the wood material. In this study we applied hyperspectral near infrared imaging to Scots pine sapwood samples exposed to the brown rot fungi *Coniophora puteana* and *Rhodonia placenta* to obtain position-resolved chemical information on the fungal degradative process. A stacked-sample decay test was used to create a succession of decay stages within the samples. The results showed that the key chemical changes associated with decay were the degradation of amorphous and crystalline carbohydrates and an increase in aromatic and carbonyl functionality in lignin. The position-resolved spectral data revealed that the fungi initiated degradation in earlywood, and that earlywood remained more extensively degraded than latewood even in advanced decay stages. Apart from differences in mass losses, the two fungi produced similar spectral changes in a similar spatial pattern. The results show that near infrared imaging is a useful tool for analyzing brown rot decayed wood and may be used to advance our understanding of fungal degradative processes.

## Introduction

Wood is a renewable biocomposite with many attractive properties, but as a natural material it is susceptible to degradation by many abiotic and biotic factors. One of the most serious forms of wood degradation is wood decay, caused by wood decaying fungi. These fungi have specialized in degrading the structural polymers that make up the wood cell walls, resulting in a loss of material and structural integrity.

Wood decaying fungi are typically classified as brown rot, white rot or soft rot fungi depending on the type of decay they cause. Although brown rot fungi make up <10% of all wood decaying fungal species (Arantes et al., 2012 and references therein), they are common degraders of structural timber (Gabriel and Švec, 2017). Brown rot fungi utilize highly destructive hydroxyl radicals derived from a biological Fenton reaction in the initial stages of decay (Arantes et al., 2012; Arantes and Goodell, 2014), causing the depolymerization of cellulose (Kleman-Leyer et al., 1992) and the depolymerization and rapid repolymerization of lignin (Yelle et al., 2008, 2011). The mechanical properties of wood deteriorate at a rate exceeding mass loss (Winandy and Morrell, 1993; Curling et al., 2002). In the following stages of decay the brown rot fungi digest the wood cell wall carbohydrates, with preferential degradation of the hemicellulose fraction (Winandy and Morrell, 1993; Curling et al., 2002). Extensively brown rotted wood consists of a brown, crumbling residue of modified lignin (Jin et al., 1990; Yelle et al., 2008, 2011).

Decay is typically quantified by determining the loss in sample mass or strength, while the effects on chemical composition are traditionally determined using wet chemical methods. Spectroscopic methods present an attractive alternative to the laborious traditional methods, and near infrared (NIR) spectroscopy in particular has been extensively utilized to study various aspects of wood chemical composition and structure. In the context of brown rot decay, NIR spectroscopy has been used to study the chemical and physical changes in wood caused by decay (Fackler et al., 2007; Fackler and Schwanninger, 2010, 2011) and as a tool to non-destructively estimate mass loss and/or strength loss due to decay (Kelley et al., 2002; Fackler et al., 2007; Green et al., 2010, 2012).

Despite the obvious usefulness of NIR spectroscopy in analyzing the effects of brown rot decay, the spectroscopic analyses fail to consider one important aspect of decay—local variations within the substrate. Wood is a highly complex cellular material, and its structure and composition vary on several length scales. These variations affect the degradative efficiency of fungi, which means that different parts of a wood sample will be degraded to different extents or at different rates (Schwarze, 2007). Fungal colonization and decay are also slow processes, resulting in further variations in the extent of decay within wood. Given these variations and the fact that different degradative reactions take place in different stages of decay, a comprehensive understanding of the wood decay process requires the collection of position-resolved chemical information from material in different stages of degradation.

The objective of this investigation was to provide an overview of the brown rot degradative process from early to advanced stages of decay. Scots pine sapwood samples were exposed to the brown rot fungi *Coniophora puteana* (Schum. ex Fries) Karst. and *Rhodonia placenta* (Fr.) Niemelä, K.H. Larsson & Schigel in a stacked-sample decay test designed to create a continuum of decay stages across the samples. Hyperspectral NIR images were collected from the decayed samples and the spectral data analyzed first by ANOVA simultaneous component analysis (ASCA) and then by principal component analysis (PCA) followed by clustering to uncover the spatial distribution of degradative reactions within the samples. The results provide a visual overview of the brown rot degradative process and demonstrate the applicability of NIR imaging to the study of wood decay.

## Materials and Methods

### Wood Samples and Decay Test

**Figure 1:**
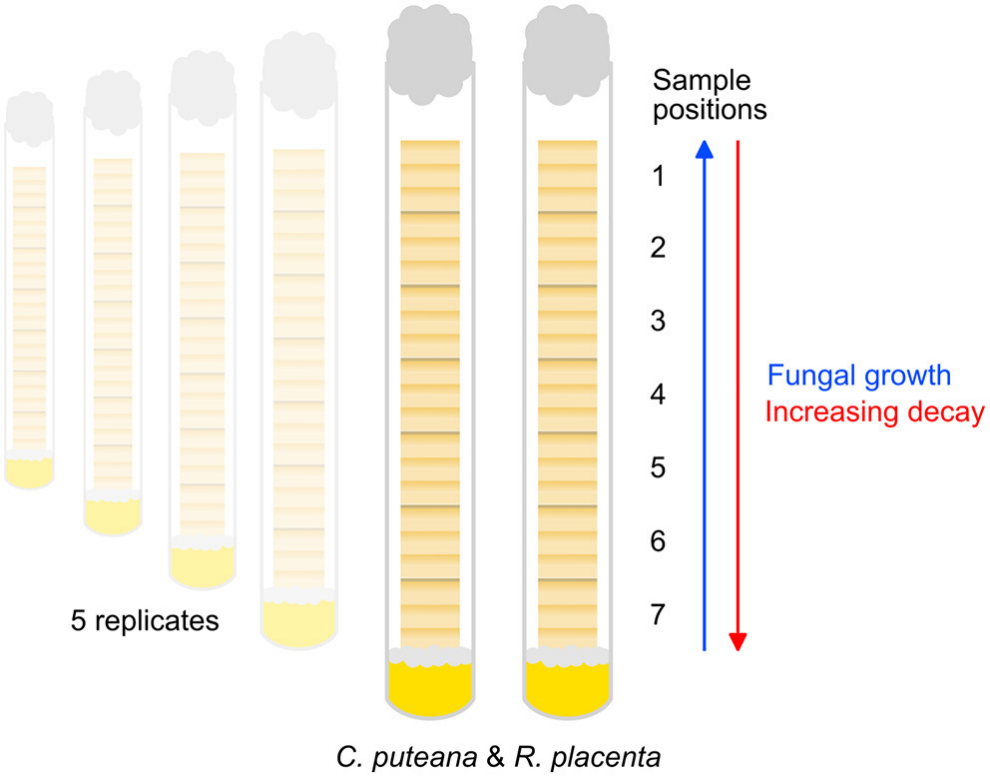
Outline of the decay test set-up. Seven samples (sample position 1–7) were stacked in test tubes over nutrient agar inoculated with *C. puteana* or *R. placenta*. The fungi colonized the samples progressively, producing a series of samples in different stages of decay.

The Scots pine sapwood samples studied in this experiment were the same as those studied in Belt et al. (2022). The samples were sized 12 × 8 × 8 mm (R × T × L) and contained 6–7 annual rings per sample, allowing the detection of potential earlywood-latewood differences and decay gradients within the samples. Before the start of the decay test the samples were dried at 60°C to determine their initial mass and then sterilized by ionizing radiation (25–50 kGy dose). The decay test (Figure 1) was conducted in 16-mm-diameter test tubes containing 4 ml of 2% malt extract agar. The tubes were inoculated with one plug of mycelium from *C. puteana* (strain BAM Ebw. 15) or *R. placenta* (strain BAM 113) stock cultures maintained on 2% malt extract agar. Seven wood samples were then added to each tube, stacked over the inoculated agar. A piece of plastic netting was placed between the inoculated agar and the first wood sample to prevent direct absorption of water from the agar. Five replicate tubes were prepared per fungal species. The tubes were plugged with cotton wool and incubated at 85% RH at room temperature. Due to the stacked arrangement of the samples in the tubes, the fungi colonized the samples successively. The decay test was allowed to continue until the visible mycelial front of one of the fungi reached the top of the topmost sample in one replicate tube. After this the samples were removed from the tubes, wiped to remove surface mycelium and then dried at 60°C to determine their decayed mass. The samples were stored in closed containers over desiccant until imaging.

### NIR Imaging

Images were collected from the radial surfaces of the decayed samples. Prior to imaging the surfaces were smoothed with a rotary microtome or, in the case of the most extensively degraded samples, by hand with a razor blade. The samples were then placed on a vertical translation stage and the height of the stage was adjusted to match each sample surface with the focal plane of the camera. Images were recorded in line scanning mode with a Specim SWIR3 (Specim, Spectral Imaging, Ltd.) short-wavelength infrared hyperspectral camera equipped with a OLES macro lens. Each line scan recorded 384 pixels and 288 spectral variables over a spectral range of 930–2,550 nm at a spectral resolution of 12 nm (full width at half maximum). The lens had a field of view of 10 mm, resulting in a nominal pixel size of ~26 × 26 μm^2^. Two halogen lamps generated polychromatic light, and a HgCdTe detector array with a grating prism monochromator gathered the reflected wavelengths from the exposed surface of the samples. A calibration reflectance target was scanned along with the samples, resulting in overall image dimensions of 1,029 × 384 pixels. All images were captured in reflectance mode.

### Image Segmentation and Transformation

The acquired images were preprocessed using a median filter with a moving window of 3 × 3 units and corrected with the measured reflectance target and dark current intensities. The reflectance images were preprocessed with standard normal variate (SNV) transformation and mean centering and the image backgrounds and saturated pixels were identified using PCA (Wold et al., 1987) and removed. One region of interest (ROI) of 281 × 301 pixels was selected from the center of each image and the ROI pixels were converted into absorbance using Equation (1):


(1) $$\begin{array}{r} \text{A}={\text{log}}_{10}\;\left(1/r\right) \end{array}$$

where A represents absorbance and r the unitless reflectance values. A ROI smaller than the sample size was used to exclude damaged regions on the edges of the most extensively decayed samples while keeping the image size consistent across all samples. Bands outside the range 1,100–2,400 nm were excluded, and the average spectra of the samples were determined based on the selected ROIs.

### ASCA

The average spectra were decomposed into the main effects and corresponding two-factor interactions between sample position, fungal species, and replicate number based on the conditions of the decay test. The decomposition was determined according to the general ASCA model (Smilde et al., 2005; Bertinetto et al., 2020, Equation 2):


(2) $$\begin{array}{r} {\text{X}}_{\text{m}}=\;{\text{X}}_{\text{p}}+\;{\text{X}}_{f}+\;{\text{X}}_{\text{r}}+\;{\text{X}}_{\left(\text{p x f}\right)}+\;{\text{X}}_{\left(\text{p x r}\right)}+\;{\text{X}}_{\left(\text{f x r}\right)}+\;\text{E} \end{array}$$

where **X**_*m*_ denotes the SNV transformed and mean centered average sample spectra, **X**_*p*_, **X**_*f*_, and **X**_*r*_ the main effect matrices of sample position, fungal species and biological replicates, respectively. The remaining effect matrices **X**_**(***p x f***)**_, **X**_**(***p x r***)**_, and **X**_**(***f x r***)**_ denoted the corresponding two-factor interactions and **E** a matrix of model residuals. Equation (2) also partitioned the variation in the spectra to their factor-specific contributions based on the corresponding sum of squares. After statistical evaluation, the decomposition in Equation (2) was revised by combining the position and the position x fungus effects (Jansen et al., 2005, Equation 3):


(3) $$\begin{array}{r} {\text{X}}_{\text{m}}=\;{{\text{X}}_{\text{p}+\left(\text{p x f}\right)}+\text{X}}_{\text{f}}+\;{\text{X}}_{\text{r}}+\;{\text{X}}_{\text{p x r}}+\;{\text{X}}_{\text{f x r}}+\;\text{E} \end{array}$$

The effect matrices in Equations (2) and (3) were then decomposed into ASCA scores and loadings (Equation 4):


(4) $$\begin{array}{r} {\text{X}}_{\text{i}}={\text{T}}_{\text{i}}{\text{P}}_{\text{i}}^{\text{T}} \end{array}$$

where **T**_*i*_ and ${\text{P}}_{i}^{T}$ **d**enoted the score and orthonormal loading (${\text{P}}_{i}^{T}{\text{P}}_{i}=\text{I}$) vectors of the effect matrix **X**_*i*_, *i* ∈ {*p*, …, *f x r*}. The natural variation within the factor levels was considered by projecting the residuals onto the ASCA loadings as discussed by (Zwanenburg et al., 2011, Equation 5):


(5) $$\begin{array}{r} {\text{S}}_{\text{i}}=\left({\text{X}}_{\text{i}}+\text{E}\right){\text{P}}_{\text{i}}={\text{T}}_{\text{i}}+\text{E}{\text{P}}_{\text{i}} \end{array}$$

where **S**_*i*_ denoted the combined ASCA scores of the effect *i*, *i* ∈ {*p*, …, *f x r*}. The statistical significance of the ASCA effects in Equation (2) were evaluated based on 10,000 permutations (Vis et al., 2007; Bertinetto et al., 2020) using the ASCA tool included in PLS toolbox (Eigenvector Research, Inc.).

### Multivariate Image Analysis

One replicate tube of samples exposed to *C. puteana* and *R. placenta* was selected for image analysis. The images collected from the selected samples were combined into one mosaic per fungal species, after which the mosaics were decomposed into PCA scores and loadings (Equation 6):


(6) $$\begin{array}{r} {\text{M}}_{\text{m}}=\text{T}{\text{P}}^{\text{T}}+\;{\text{E}}_{\text{n}} \end{array}$$

where **M**_*m*_ denotes the preprocessed and mean centered mosaic spectra, **T** a matrix of PCA score vectors, **P** a matrix of PCA loadings (**P**^*T*^**P=I**), and **E**_*n*_ a residual matrix after *n* principal components. The calculations were performed on unfolded mosaics using the singular value decomposition algorithm (Wall et al., 2003). The first principal component was used to remove extreme pixels based on threshold values and the PCA scores and loadings were redetermined. The final scores were refolded back into mosaic dimensions to visualize the chemical changes and to interpret the respective loadings.

Finally, a PCA-based clustering approach was used to group the image pixels. K-means clustering segregated the pixels into distinct classes based on their correlation with the mean of each cluster. Euclidean distances were used to select the first centroids furthest away from the center of the score space. The average class spectra were determined and the class vectors were refolded back into mosaic dimensions. The data analyses were performed using in-house Matlab® scripts (MathWorks, Inc.) including commercial functions from the PLS toolbox (Eigenvector Research, Inc.).

## Results

### Decay Test Results

**Figure 2:**
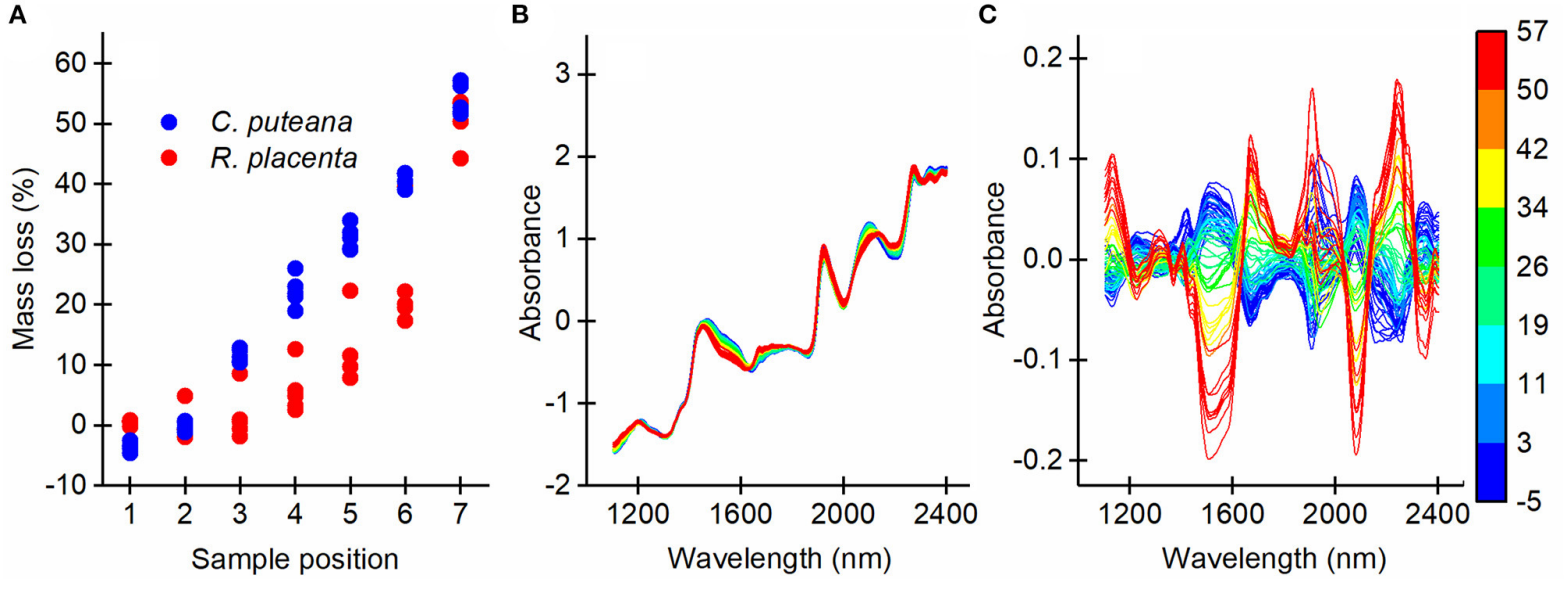
Mass losses of all sample blocks caused by *C. puteana* and *R. placenta* **(A)** and the SNV transformed average NIR spectra of the samples before **(B)** and after mean-centering **(C)** colored according to mass loss.

To produce a series of samples in different stages of brown rot decay, samples of Scots pine sapwood were exposed to pure cultures of *C. puteana* and *R. placenta* in a stacked-sample decay test. The mass losses of the samples were measured, after which the samples were analyzed by NIR imaging to gather information on the chemical changes caused by decay and their spatial distribution within the samples. The mass loss measurements (Figure 2A) showed that the decay test succeeded in producing a succession of decay stages. The samples exposed to *C. puteana* exhibited a linear increase in mass loss from an average of −3.6% at position 1 (topmost sample) to an average of 54.0% at position 7 (bottommost sample). *R. placenta* produced a non-linear increase in mass loss, reaching an average of 50.8% at position 7. Apart from the high mass losses recorded at position 7, the mass losses caused by *R. placenta* were substantially lower than their *C. puteana* counterparts. The SNV transformed and mean-centered average NIR spectra of the samples (Figure 2C) indicated substantial chemical changes in the samples as a function of mass loss.

### ASCA

**Figure 3:**
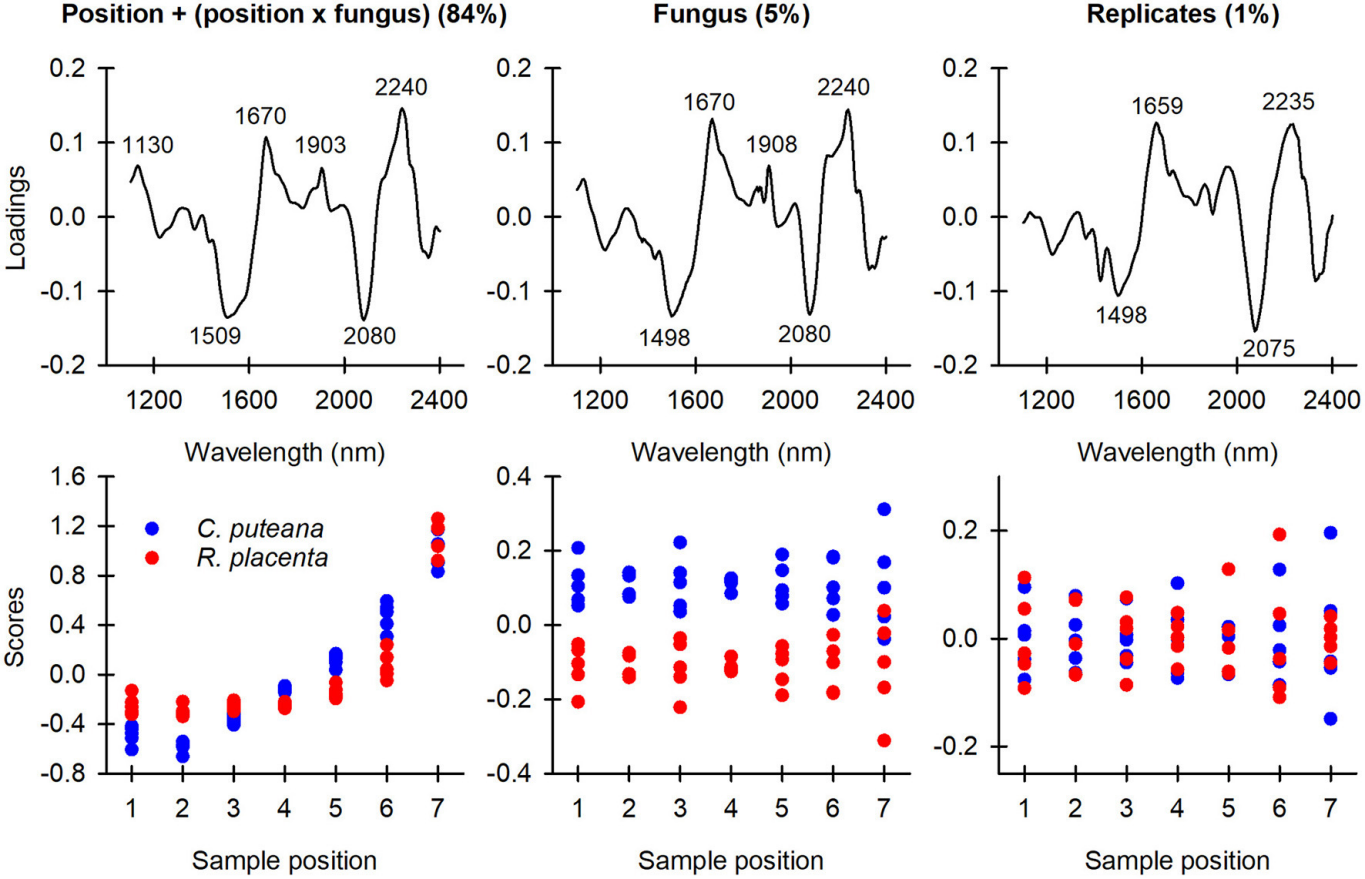
ASCA first component loadings and scores of the position + (position × fungus) combination effect, the fungus effect, and the replicate effect.

The spectral data were first analyzed by ASCA to identify the key spectral changes caused by decay and to look for differences between the two fungal species. As shown in Table 1, the position and fungus main effects and the position × fungus interaction were found to have a statistically significant effect (*p* < 0.05 according to permutations) on the variation in the sample spectra. The position and the position × fungus interaction effects were then combined into one effect. This step enabled us to include the significant position effects into a single sub-model while still isolating the main fungus effect. The ASCA loadings and scores of the first component of the fungus effect and the combined position + (position × fungus) effect are shown in Figure 3, along with the ASCA loadings and scores of the replicate effect. There was no consistent pattern in the replicate effect scores, which showed that there were no systematic differences between the replicates.

**Table 1:** The variation and significance of the main effects and their two-factor interactions from ASCA.

**Effect**	**Position**	**Fungus**	**Replicate**	**Position × fungus**	**Position × replicate**	**Fungus × replicate**	**Residual**
Variation (%)	75.7	4.3	1.3	8.1	4.6	0.7	5.2
*p*-value^a^	<0.01	<0.01	0.40	<0.01	0.71	0.67	–

a *Based on 10,000 permutations*.

The position + (position × fungus) combination effect accounted for 84% of the data variation. The loading had positive bands at 1,130, 1,670, 1,903 and 2,240 nm, and negative bands at 1,509 and 2,080 nm. The bands at 1,130 and 1,670 nm were assigned to the aromatic C-H stretch of lignin (Shenk et al., 1992; Fackler and Schwanninger, 2010; Sandak et al., 2011; Schwanninger et al., 2011a), while the band at 1,903 nm was assigned to the C=O stretch of lignin (Schwanninger et al., 2011b; Popescu and Popescu, 2013). The positive bands at 1,509 and 2,080 nm are due to the O-H stretch of cellulose interchain hydrogen bonds and the O-H stretch + C-H deformation of semi-crystalline or crystalline cellulose, respectively (Watanabe et al., 2006; Fackler and Schwanninger, 2010; Schwanninger et al., 2011a). The position + (position × fungus) effect scores changed from negative to positive from sample position 1 to position 7 for both fungal species, indicating a decrease in cellulose content and an increase in lignin content, particularly in aromatic and carbonyl functionalities. The score values and gravimetrically determined mass losses (Figure 2) followed similar trends as a function of mass loss.

The fungus effect explained 5% of the total data variation. The loading vector was very similar to the position + (position × fungus) combination effect loading, with positive bands at 1,670, 1,908, and 2,240 nm (derived from lignin) and negative bands at 1,498 and 2,080 nm (derived from cellulose). The *C. puteana*-degraded samples had more positive effect scores at every sample position than the *R. placenta*-degraded samples, suggesting that the samples attacked by *R. placenta* had a higher carbohydrate content and lower lignin content than the *C. puteana* samples. This difference in composition is likely to be primarily due to the higher mass losses of the *C. puteana*-exposed samples. However, the score value differences between the two fungi persisted even at sample position 7 where the mass loss differences were small (Figure 2), which suggests that the spectral differences are not solely a function of mass loss. In an effort to identify chemical differences independent of mass loss between the two species, the loadings of the fungus effect and the position + (position x fungus) effect were compared (see Supplementary Figure 1). The fungus effect loading showed deviations from the combined effect loading at 1,404, 1,591, 1,887, 2,020, and 2,158 nm. The 1,404, 1,591, and 1,887 nm spectral contributions were assigned to phenolic OH groups, crystalline cellulose and lignin carbonyl groups (Tsuchikawa and Siesler, 2003; Schwanninger et al., 2011a; Popescu and Popescu, 2013), respectively, which suggests that differences related to lignin modification and cellulose degradation exist between the fungi.

### PCA

**Figure 4:**
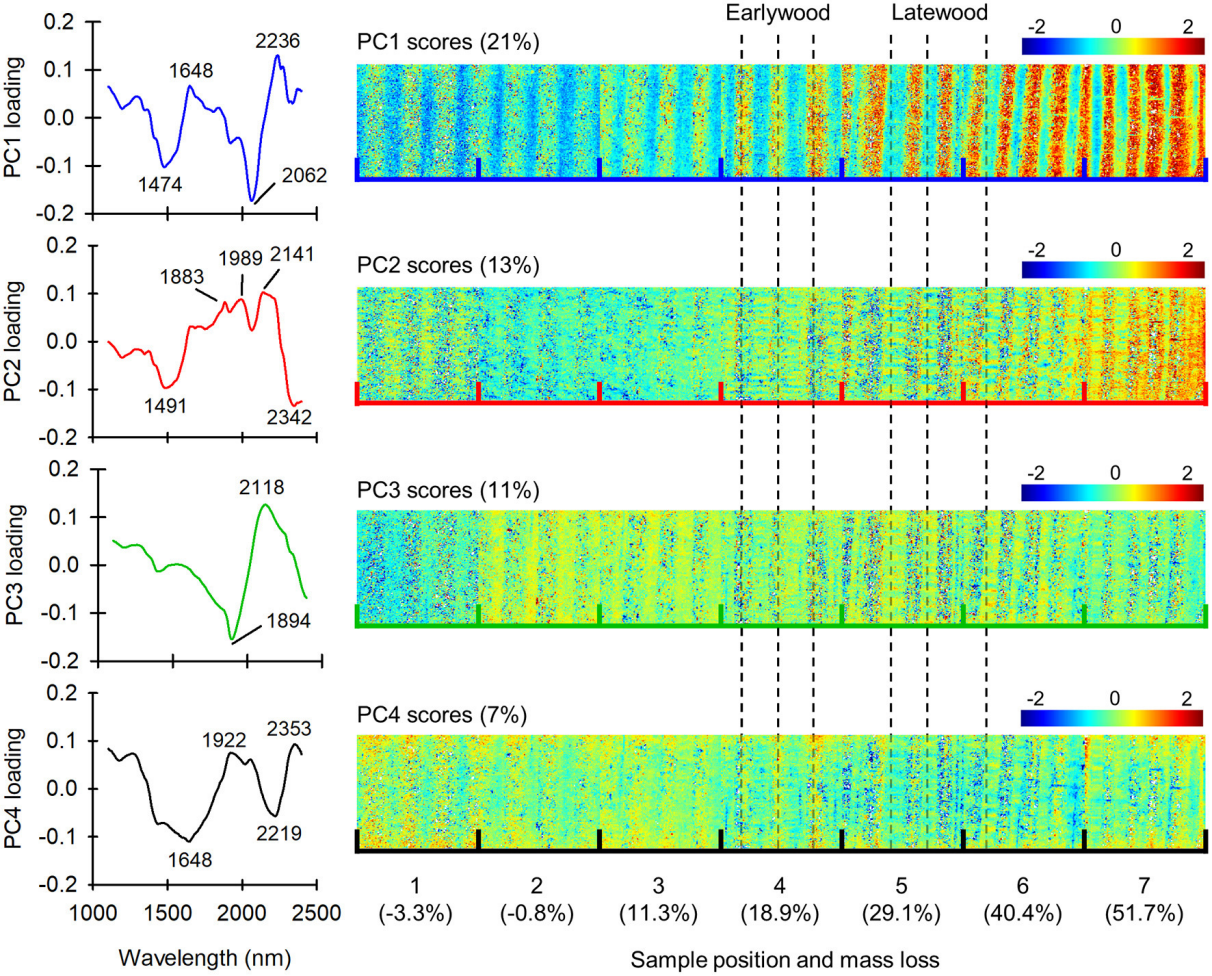
Loadings and scores of PCs 1–4 in a set of samples exposed to *C. puteana*. Each individual image in the score mosaics is sized 7.3 × 7.8 mm.

ASCA is an effective method for separating the effects of different variables on the overall data variation, but it is not ideal for imaging data due the averaging involved in determining the ASCA effects. Therefore, to obtain position-resolved information on decay progression, one sample tube per fungal species with mass losses close to the fungus-specific mean was selected and the images analyzed by PCA. Figure 4 shows the loadings and scores of the first four PCs derived from the *C. puteana* sample set.

PCs 1 and 2 explained 21 and 13% of the data variation, respectively, and provided information related to fungal degradation and earlywood-latewood differences. The PC1 loading vector had positive bands at 1,648 and 2,236 nm and negative bands at 1,474 and 2,062 nm. The 1,648 nm band was assigned to the C-H stretch of aromatic groups in lignin, while the 1,474 and 2,062 nm bands were assigned to the O-H stretch of glucomannan or amorphous/semi-crystalline cellulose, and the O-H stretching + O-H deformation of semi-crystalline/crystalline cellulose, respectively (Sandak et al., 2011; Schwanninger et al., 2011a). The PC2 loading vector had positive bands at 1,883, 1,989, 2,141 nm and negative bands at 1,491 and 2,342 nm. The positive bands at 1,883 and 2,141 nm were assigned to the C=O and C-H + C=O stretch of lignin (Popescu and Popescu, 2013), while the negative 1,491 nm band was assigned to the O-H stretch of glucomannan or amorphous/semi-crystalline cellulose (Tsuchikawa and Siesler, 2003; Schwanninger et al., 2011a). The 2,342 nm band was derived from cellulose (Shenk et al., 1992; Schwanninger et al., 2011a). Both the PC1 and PC2 scores increased from sample position 1 to position 7, reflecting selective carbohydrate degradation. Earlywood and latewood regions were identified by visual inspection of false-color images generated from unprocessed image data, and it was found that positive PC1 scores were associated primarily with earlywood, while positive PC2 scores were in turn more strongly associated with latewood. This suggests that the degradation of earlywood is characterized by strong degradation of both low and high crystallinity carbohydrates and an increase in aromatic lignin content, while the degradation of latewood is characterized by the degradation of low crystallinity carbohydrates and an increase in lignin carbonyl content.

PCs 3 and 4 explained 11 and 7% of the data variation, respectively. The PC3 loading vector had a positive band at 2,118 nm assigned to cellulose (Schwanninger et al., 2011a) and a negative band at 1,894 assigned to the C=O stretch of lignin (Popescu and Popescu, 2013). The PC3 scores were generally more positive in latewood than earlywood and decreased from sample position 2 to position 7, indicating a decrease in cellulose content and an increase in lignin carbonyl content, particularly in earlywood. The PC4 loading vector in turn had positive bands at 1,922 and 2,353 nm and negative bands at 1,648 nm and 2,219 nm. The PC4 scores were more positive in earlywood at sample positions 1–4 and in latewood at position 5–7, and they generally decreased from position 1 to position 7. The overall decrease in PC4 scores from position 1 to position 7 is consistent with a decrease in cellulose content and an increase in lignin content.

The loadings and scores of the first four PCs derived from the *R. placenta* sample set are given in Supplementary Figure 2. The *R. placenta* set yielded results similar to the *C. puteana* set, with PC1 (25%) and PC2 (13%) providing information on carbohydrate degradation and lignin modification in earlywood and latewood, respectively. PC3 (8%) and PC4 (6%) were also similar to those derived from the *C. puteana* sample set, although the scores did not follow a consistent trend as a function of sample position.

### Clustering

**Figure 5:**
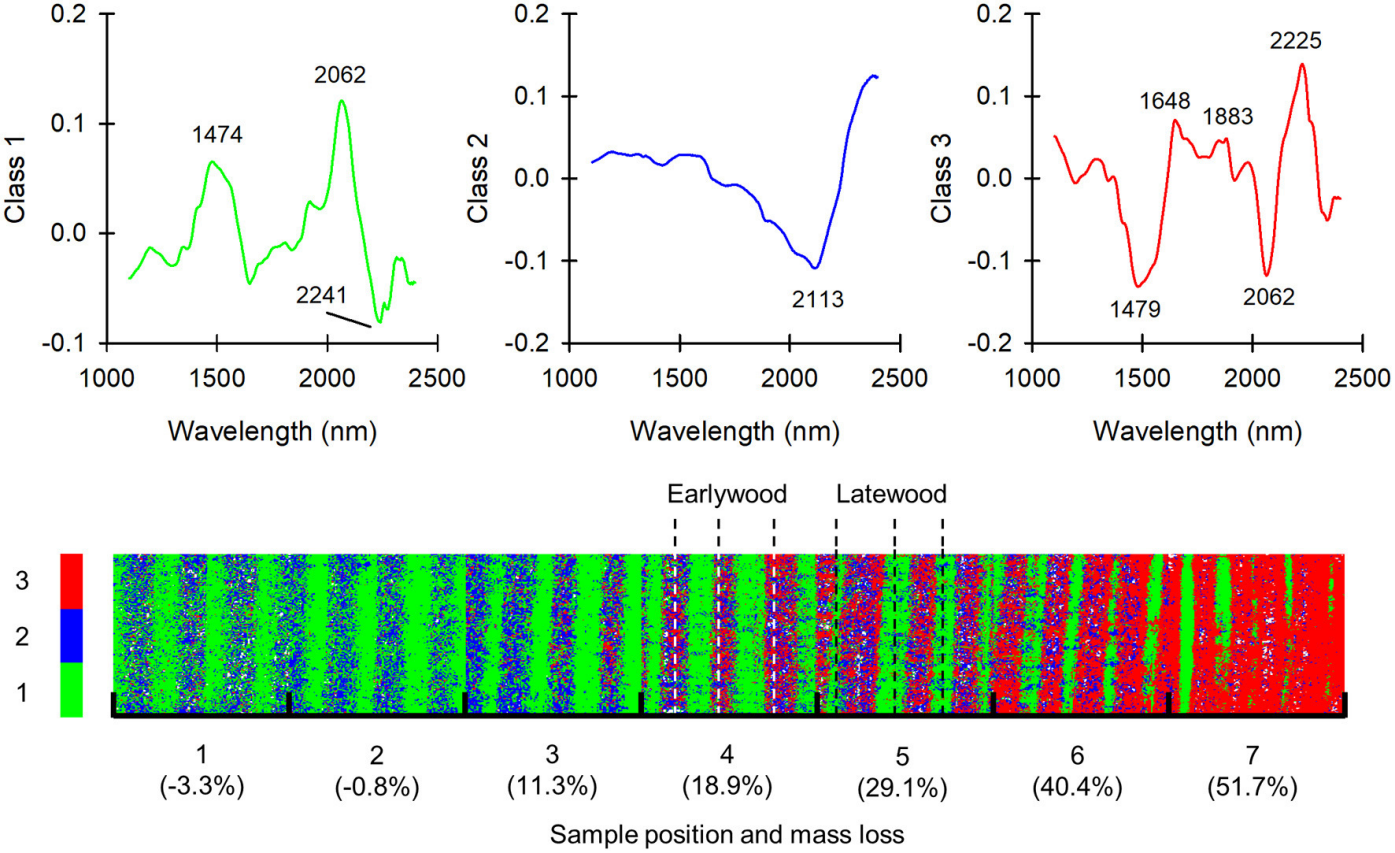
Mean-centered spectra of classes 1–3 and the class assignments of image pixels in a set of samples exposed to *C. puteana*. Each individual image in the mosaic is sized 7.3 × 7.8 mm.

To get a clearer view of the progress of degradation in the samples, the spectra in the *C. puteana* and *R. placenta* data sets were separated into distinct classes using k-means clustering based on the scores of PCs 1–4. The number of classes was set to three as this was found to provide the clearest separation between degraded and undegraded areas. The mean-centered spectra of the three classes and the class assignments of the image pixels are given in Figure 5 for the *C. puteana*-degraded sample set.

The mean-centered spectrum of class 1 had carbohydrate-derived positive bands at 1,474 nm (glucomannan or amorphous/semi-crystalline cellulose) and 2,062 nm (semi-crystalline/crystalline cellulose), along with a negative band at 2,241 nm. The mean-centered spectrum of class 2 in turn showed only a broad negative spectral feature at 2,113 nm assigned to cellulose (Schwanninger et al., 2011a). The mean-centered spectrum of class 3 showed features of brown rot degradation: it had lignin-derived positive bands at 1,648 nm (aromatic groups), 1,883 nm (C=O) and 2,225 nm, and carbohydrate-derived negative bands at 1,479 nm (glucomannan or amorphous/semi-crystalline cellulose) and 2,062 nm (semi-crystalline/crystalline cellulose). In the least degraded samples at positions 1 and 2, the latewood regions of the samples were represented by the carbohydrate-rich class 1, while the earlywood regions were assigned to a mixture of class 1 and class 2. The assignment of some earlywood pixels to class 2 was interpreted as a representation of incipient brown rot decay, although it may also be at least partially due to the lower carbohydrate content of undegraded earlywood (Bertaud and Holmbom, 2004). Further signs of brown rot degradation appeared in earlywood at sample position 3, where the pixels assigned to the carbohydrate-rich class 1 disappeared while pixels assigned to the degradation-specific class 3 appeared. At position 7 virtually all of the earlywood pixels were assigned to class 3. The first signs of latewood degradation were seen at sample positions 4 and 5, where pixels assigned to class 2 appeared amongst the class 1 pixels. At position 6 the width of the intact latewood regions represented by class 1 appeared to decrease, which suggests that the latewood regions were progressively degraded, presumably from the earlywood side of the region. Most of the latewood regions were degraded and represented by class 3 at position 7, although narrow bands of intact latewood still persisted.

The application of clustering to the *R. placenta*-degraded sample set provided similar results (see Supplementary Figure 3). In the least degraded samples at positions 1 and 2, the latewood regions of the samples were represented by the carbohydrate-rich class 1, while the earlywood regions were represented by class 2. Brown rot degradation became apparent at sample positions 3 and 4, where pixels represented by the degradation-specific class 3 appeared in the earlywood regions. Latewood degradation became apparent only at position 1, where most of the image pixels were assigned to class 3. Thin bands of intact latewood represented by class 1 could be seen at the less degraded end of the sample at position 1.

## Discussion

Analysis of the spectral data by ASCA and PCA showed that both *C. puteana* and *R. placenta* caused carbohydrate degradation and lignin modification in Scots pine sapwood in the decay test set-up utilized in this experiment. The ASCA results (Figure 3) revealed that the main spectral changes associated with decay were the degradation of semi-crystalline and crystalline cellulose and the increase in aromatic and carbonyl functionality in lignin. PCA identified similar changes (Figure 4 and Supplementary Figure 2). Cellulose degradation is a typical finding in brown rot decay as the fungi mineralize only the carbohydrate fraction of the wood cell wall. Brown rot fungi usually digest hemicelluloses ahead of cellulose (Winandy and Morrell, 1993; Curling et al., 2002), and the identification of cellulose rather than hemicellulose degradation as a key spectral change due to decay is most likely a consequence of the high mass losses seen in the more extensively degraded samples. The increasing contributions from carbonyl and aromatic functions in lignin are in turn consistent with lignin modification along with an overall increase in lignin content. Brown rotted lignin has been shown to have increased carbonyl and aromatic content, in addition to increased hydroxyl and reduced methoxyl content (Jin et al., 1990; Yelle et al., 2008, 2011). Lignin modification is thought to be caused by hydroxyl radicals, which are produced by brown rot fungi in the initial stages of decay (Arantes et al., 2012; Zhang et al., 2016; Zhang and Schilling, 2017). Hydroxyl radical attack causes the depolymerization and rapid repolymerization of lignin (Yelle et al., 2008, 2011), resulting in structural arrangements that allow the digestion of the carbohydrate fraction of the cell walls. The carbohydrates are enzymatically hydrolyzed into sugars after the initial radical attack (Zhang et al., 2016; Zhang and Schilling, 2017), either in the cell wall after the structure has been opened up by radical attack, or more likely in the cell lumen after diffusion of radical-generated carbohydrate fragments into the lumen (Goodell et al., 2017).

Analysis of the image-form data by PCA (Figure 4 and Supplementary Figure 2) showed that the earlywood regions of the brown rot degraded samples were characterized by the degradation of both high and low crystallinity carbohydrates and an increase in lignin carbonyl and aromatic content, while the latewood regions were characterized by the degradation of low crystallinity carbohydrates and an increase in lignin carbonyl content. Although the spectral differences between earlywood and latewood may reflect some variations in the fungal degradative process between earlywood and latewood, the differences are most likely primarily derived from differences in the extent of decay. The fact that brown rot fungi degrade amorphous carbohydrates ahead of crystalline cellulose (Winandy and Morrell, 1993; Curling et al., 2002) suggests that the earlywood regions showing high-crystallinity and low-crystallinity carbohydrate degradation are more extensively degraded than the latewood regions, which showed only low crystallinity carbohydrate degradation. The association of increasing carbonyl content with latewood also supports this, given that lignin modification is thought to take place in the initial decay stages. The association of increasing aromatic content in addition to increasing carbonyl content with earlywood is most likely related to the increasing residual lignin content with selective carbohydrate removal.

PCA and cluster analysis showed that both *C. puteana* (Figures 3, 4) and *R. placenta* (Supplementary Figures 2, 3) initiated degradation in the earlywood regions of the samples and that the earlywood regions remained more extensively degraded even in advanced decay. Brown rot decay is known to be initiated in earlywood where the large cell lumens and abundant pit connections allow the fungal hyphae to spread (Schwarze, 2007; Bouslimi et al., 2014). Microscopy-based studies have demonstrated that earlywood shows more visual signs of decay during the incipient stages (Schwarze, 2007; Bouslimi et al., 2014), but X-ray densitometry results have shown that both the absolute and relative density losses of latewood quickly surpass the density losses of earlywood as the decay progresses (Bucur et al., 1997; Macchioni et al., 2007; Reinprecht et al., 2007). We did not perform density measurements, but our results demonstrate that in terms of chemical composition, earlywood remains more extensively degraded than latewood until very advanced decay stages.

Apart from differences in the mass losses recorded at different sample positions in the decay test tubes (Figure 2), our results showed that *C. puteana* and *R. placenta* produced highly similar degradative changes in pine sapwood. Genome sequencing and gene expression analyses have revealed notable differences in degradative machinery between *C. puteana* and *R. placenta* (Floudas et al., 2012), but our results showed that the two fungi produced similar spectral changes related to carbohydrate degradation and lignin modification and similar spatial patterns of decay involving extensive earlywood degradation. Fackler and Schwanninger (2010) were unable to detect meaningful NIR spectral differences between wood degraded by *R. placenta* and the brown rot fungus *Gloeophyllum trabeum*, and strong similarities in the physiochemistry of degraded wood have been observed after degradation by brown rot fungi of distinct evolutionary origins (Kaffenberger and Schilling, 2015). Comparison of the ASCA loadings of the fungus main effect and the position + (position × fungus) combination effect (Supplementary Figure 1) revealed differences related to crystalline cellulose and hydroxyl and carbonyl groups on lignin, which may reflect chemical differences in the wood degraded by the two fungi. However, a more comprehensive assessment of the chemical differences would require the analysis of a series of samples showing comparable mass losses.

## Conclusions

NIR hyperspectral imaging in combination with ASCA, PCA and clustering showed that the degradation of pine sapwood by *C. puteana* and *R. placenta* resulted in carbohydrate degradation and lignin modification. Consistent with the current understanding of brown rot decay, our results showed that both fungi initiated degradation in earlywood. However, our results also demonstrated that the earlywood regions of the samples remained more extensively degraded even in advanced decay. Apart from differences in the recorded mass losses, the degradative changes caused by *C. puteana* and *R. placenta* were very similar. Our results showed that NIR hyperspectral imaging is a suitable method for obtaining position-resolved chemical information from brown rot decayed samples, making it a useful tool for understanding the fungal degradative process. In the future the method may be used to examine the degradation of different types of wood materials such as modified wood for which the fungal degradative process is less well-understood.

## Data Availability Statement

The datasets presented in this study can be found in online repositories. The names of the repository/repositories and accession number(s) can be found at: 10.5281/zenodo.6616006.

## Author Contributions

TB conceived and designed the study and performed the decay test. TB and MA collected the NIR images. MA and MM processed the spectral data and performed the multivariate data analyses. TB wrote the manuscript with contributions from all co-authors. All authors approved the manuscript.

## Funding

This work received funding from the Academy of Finland (grant no. 330087).

## Conflict of Interest

MM was employed by VTT Technical Research Centre of Finland Ltd. The remaining authors declare that the research was conducted in the absence of any commercial or financial relationships that could be construed as a potential conflict of interest.

## Publisher's Note

All claims expressed in this article are solely those of the authors and do not necessarily represent those of their affiliated organizations, or those of the publisher, the editors and the reviewers. Any product that may be evaluated in this article, or claim that may be made by its manufacturer, is not guaranteed or endorsed by the publisher.
