# Supplementary material for: Chemical Characterization and Visualization of Progressive Brown Rot Decay of Wood by Near Infrared Imaging and Multivariate Analysis
Source: Front Plant Sci. 2022 Jul 12;13:940745. doi: 10.3389/fpls.2022.940745 (PMC9315348; doi:10.3389/fpls.2022.940745)
Supplement: Supplementary file 1 [file Data_Sheet_1.PDF]

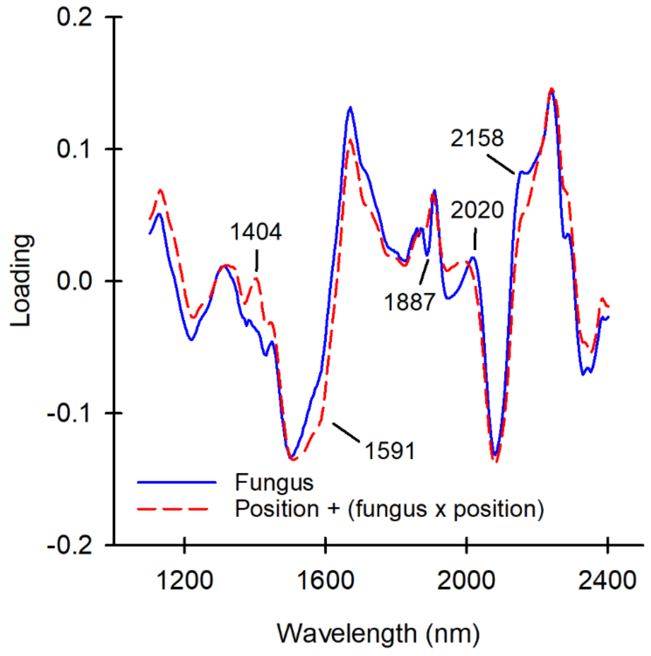

Supplementary Figure S1. ASCA first component loadings of the fungus effect and the position + (position x fungus) combination effect

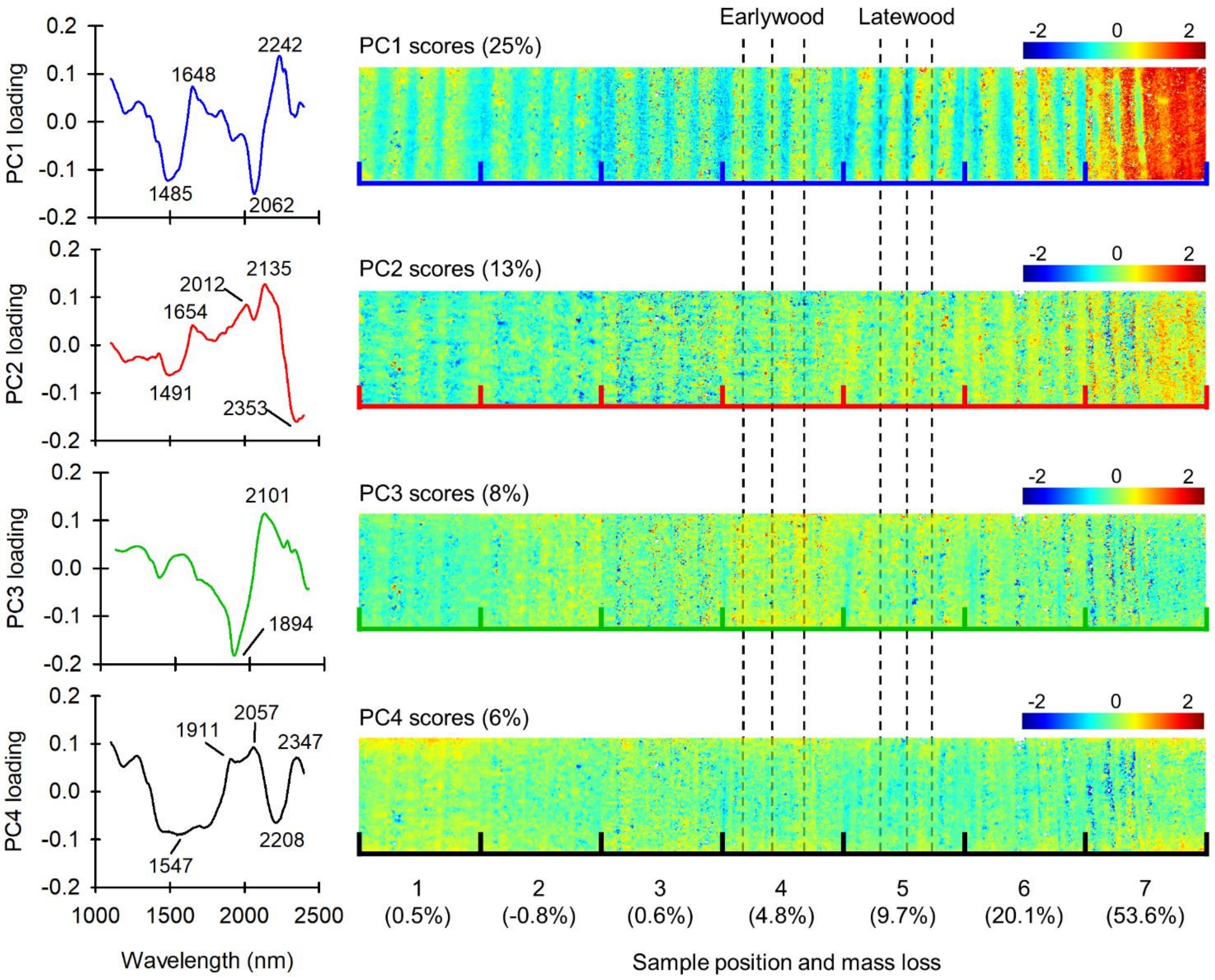

Supplementary Figure S2. Loadings and scores of PCs 1-4 in a set of samples exposed to *R. placenta*

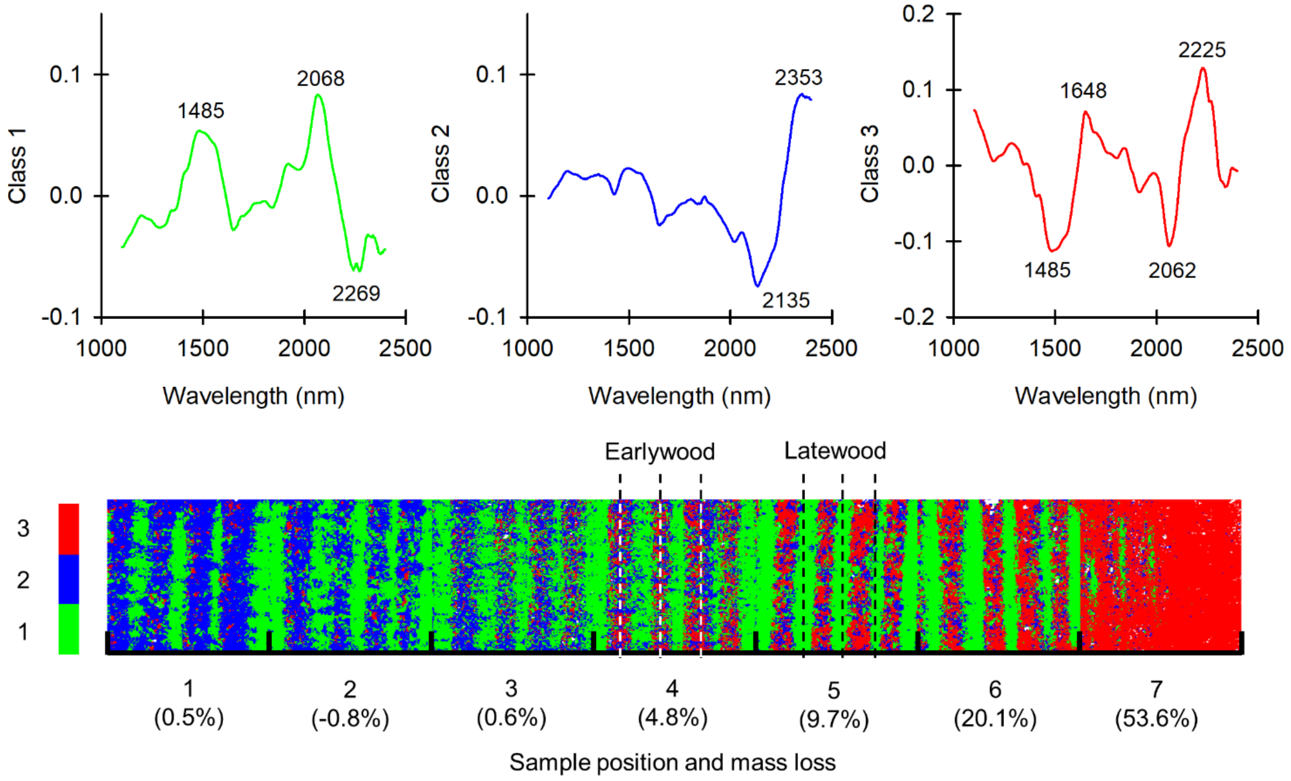

Supplementary Figure S3. Mean-centred spectra of classes 1-3 and the class assignments of image pixels in a set of samples exposed to *R. placenta*
